# A 40–50 GHz RF Front-End with Integrated Local Oscillator Leakage Calibration

**DOI:** 10.3390/mi14112105

**Published:** 2023-11-16

**Authors:** Peigen Zhou, Pinpin Yan, Jixin Chen, Zhe Chen, Wei Hong

**Affiliations:** State Key Laboratory of Millimeter Waves, School of Information Science and Engineering, Southeast University, Nanjing 210096, Chinaweihong@seu.edu.cn (W.H.)

**Keywords:** detection mixer, front-end, LO leakage calibration, SiGe, TX

## Abstract

This article presents a transmitter (TX) front-end operating at frequencies covering 40–50 GHz, including a differential quadrature mixer with integrated amplitude and phase imbalance tuning, a power amplifier, and a detection mixer (DM) that supports local oscillator (LO) leakage signal or image signal calibration. Benefiting from the amplitude and phase imbalance tuning network of the in-phase quadrature (IQ) signal generator at the LO input, the TX exhibits more than 30 dBc image signal rejection over the full frequency band without any post-calibration. Based on the LO leakage signal fed back by the DM integrated at the RF output, the LO leakage of the TX has been improved by more than 10 dB through the LO leakage calibration module integrated in the quadrature mixer. When the intermediate frequency (IF) signal is fixed at 1 GHz, the TX’s 1 dB compressed output power (OP1 dB) is higher than 13.5 dBm over the operating band. Thanks to the LO leakage signal calibration unit and the IQ signal generator, the TX is compliant with the error vector magnitude (EVM) requirement of the IEEE 802.11aj standard up to the 64-quadrature amplitude modulation (QAM) operating mode.

## 1. Introduction

The main ways to increase the communication rate are to increase the modulation order or adopt a wider channel bandwidth. Due to the limited spectrum resources in the microwave frequency band, in order to improve the communication rate, it is necessary to use a higher modulation order to improve the spectrum utilization, but it is still difficult to achieve a data rate of tens of Gbps [1,2,3,4,5]. Therefore, facing the development requirements of high-speed wireless communication in the future, it is inevitable to develop millimeter wave frequency bands with abundant spectrum resources [6,7,8]. Traditional millimeter wave discrete devices are expensive and have large board-level interconnect losses between devices. The independent design, discrete implementation and mechanical assembly of millimeter wave functional modules can no longer meet the requirements of future mobile communication systems for cost, power consumption and integration. Therefore, the study of a silicon-based highly integrated RF front-end is the key to future millimeter wave wireless communication systems.

However, in terms of system implementation and application, millimeter wave frequency bands face some inherent difficulties. First, the path loss of free space propagation increases exponentially with the increase in frequency, and secondly, atmospheric and rainwater attenuation are frequency selective, even if there is an atmospheric window at some frequency points, but the overall attenuation trend is positively correlated with frequency [9]. This makes wireless signal transmission in millimeter wave bands require higher output power or effective isotropic radiated power (EIRP) to combat spatial loss. Limited by the bias voltage of the silicon-based process, considering the system’s heat dissipation and DC-to-RF conversion efficiency, even if the technical scheme of on-chip multiplexed power synthesis is adopted, the linear output power of a single radio frequency (RF) channel in the millimeter wave band is limited, and it is still less than 20 dBm within 50 GHz. An effective solution is to use a phased array architecture with multichannel spatial power synthesis to effectively boost the EIRP of the transmitter [10,11,12]. In order to perform spatial synthesis efficiently, it is necessary to integrate amplitude and phase tuning units on each channel for analog multi-beam architecture, and the complexity and chip size are usually large. For all-digital multi-beam architectures, RF front-ends typically do not need to integrate a phase-shifter and variable gain amplifier with large size and power consumption, because amplitude and phase tuning are completed at the baseband [3].

Zero-IF transmitters are widely used in the design of millimeter wave transceivers due to their strong anti-interference ability, large bandwidth, and ability to cope with diversified scene requirements [13,14]. Although zero-IF TXs simplify the architecture of the system, implementing a zero-IF RF front-end presents the problem of insufficient suppression of image signals and LO leakage signals, especially when the operating frequency rises to the millimeter wave band. When the image frequency signal and LO leakage signal are poorly suppressed, the modulation order that the RF front-end can support will be reduced, which will greatly affect the transmission data rate that the TX can support.

In this work, we present an RF front-end with a zero-IF architecture. In the IQ signal generator in the quadrature mixer, the introduction of amplitude and phase imbalance tuning networks ensures good amplitude-phase balance of the IQ signal in the 40–50 band, and it effectively improves the suppression of the image signal of the zero-IF TX. In addition, for the LO leakage signal in the zero-IF TX, based on the LO power level fed back by the RF output detection mixer, the tail current source integrated in the quadrature mixer is tuned, which greatly improves the LO leakage signal suppression of the TX. Benefiting from the designed image signal and LO leakage signal calibration unit, the suppression of the LO leakage signal and image signal of the zero-IF TX in the working frequency band exceeds 30 dBc without integrating a phase shifter and variable gain amplifier, which are sufficient to support QAM-64 modulation of the IEEE 802.11aj standard [7], and the supported transmission data rate can exceed 30 Gbps. This miniaturized zero-IF RF front-end without an integrated phase shifter and variable gain amplifier offers significant advantages in an all-digital multi-beam phased array architecture.

This paper is organized as follows. In Section 2, we will introduce the architecture of the front-end. The circuits design methodology are presented in Section 3. The measurement results are provided in Section 4. Finally, conclusions are drawn in Section 5.

## 2. Architecture of the Front-End

**Figure 1:**
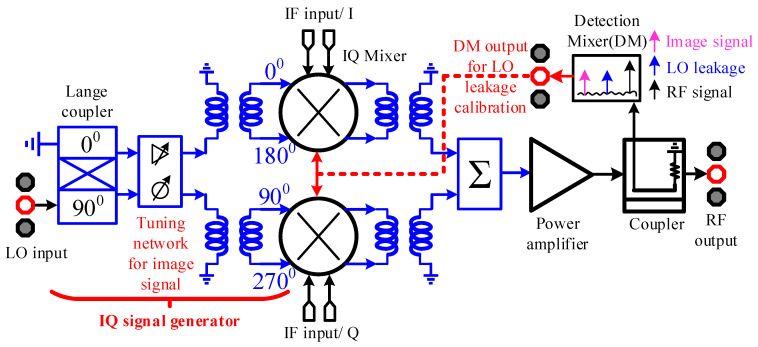
Block diagram of the 40–50 GHz front-end.

Figure 1 shows the system block diagram of the zero-IF TX, including a differential quadrature mixer that supports image signal tuning, an on-chip power synthesizer, a power amplifier, an RF coupler, and a detection mixer. The single-ended LO input signal is converted into a differential IQ signal by an on-chip IQ signal generator, which serves as the LO input signal of the image-suppressed quadrature mixer. Based on the traditional IQ signal generator, an on-chip amplitude and phase tuning network is introduced to ensure the balance of IQ signals in the 40–50 frequency band. The differential outputs of the two mixers are first converted to a single-ended signal by the on-chip transformer balun, and then the upper sideband is extracted by the on-chip power synthesizer. The output of the quadrature image rejection mixer is connected to an on-chip power amplifier to effectively increase the output power of the TX. An on-chip passive coupler is connected between the output of the power amplifier and the RF output PAD with a coupling degree of about 15. The coupling port of the coupler is connected to a detection mixer, and by mixing the RF output signal with the image frequency signal or the LO leakage signal, the amplitude of the image frequency signal or the LO leakage signal can be judged by the IF signal obtained by down-conversion. The calibration of the LO leakage can be completed by using the IF output information of the detection mixer combined with the tunable tail current source in the quadrature mixer.

## 3. Circuit Design Methodology

In this part, we will introduce the design methodology of the 40–50 GHz TX front-end, including the consideration of each block, and then present the design methodology of key building blocks.

### 3.1. Detection Mixer

**Figure 2:**
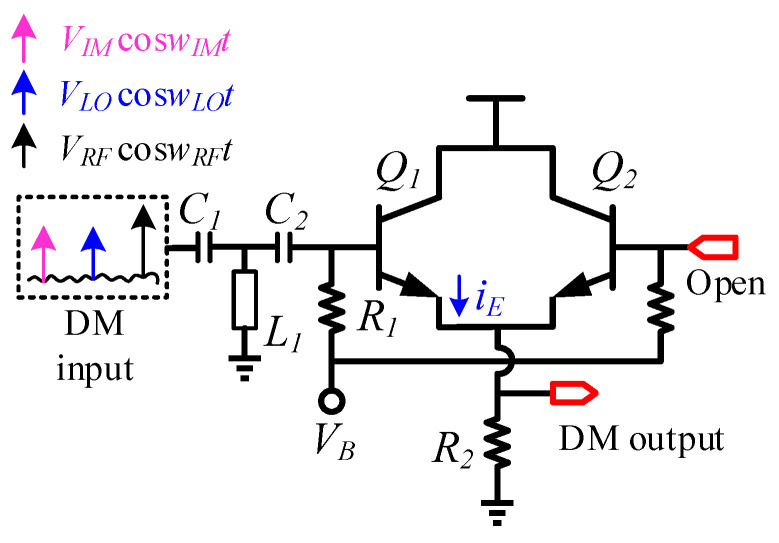
Schematic of the detection mixer.

The circuit schematic of the detection mixer is shown in Figure 2. The input of the detection mixer is connected to the coupling port of the coupler, and the input signal mainly includes RF signal *V_RF_* cos*w_RF_t*, LO leakage signal *V_LO_* cos*w_LO_t* and image frequency *V_IM_* cos*w_IM_t* signal. The input impedance matching network of the detection mixer consists of a wideband T-network consisting of two series capacitors *C*_1_, *C*_2_ and an inductor *L*_1_ connected in parallel to RF ground. Transistor *Q_1_* with a common collector structure is used as the detection mixing core [15]. Signals *V_RF_* cos*w_RF_t*, *V_LO_* cos*w_LO_t*, and *V_IM_* cos*w_IM_t* are injected through the base of the mixing core. Transistor *Q_2_* and transistor *Q*_1_ form a symmetrical structure, and the base of transistor *Q*_2_ is in a suspended state, which is mainly used to improve the robustness of RF performance of transistor *Q*_1_ with changes in temperature, voltage, process angle, etc. [16,17]. The IF signal of the detection mixer is AC-coupled through the emitter of transistor *Q*_1,2_. For this zero-IF TX, the image signal power at the RF output is much smaller than the LO leakage signal due to the image rejection considered in the design of the differential quadrature mixer. Therefore, this detection mixer is mainly used to complete the calibration of the LO leakage signal.

For RF signal *V_RF_ cosw_RF_t* and LO leakage signal *V_LO_ cosw_LO_t* at the input of the detection mixer, the amplitude of the RF signal is much higher than the amplitude of the LO leakage signal due to the quadrature image suppression mixing architecture, that is

(1) $${\;V}_{RF}\gg V_{LO}$$

For the detection mixer core shown in Figure 2, let

(2) $$v_{RF}\left(t\right)=V_{RF}cosw_{RF}t$$

(3) $$v_{LO}\left(t\right)=V_{LO}cosw_{LO}t$$

when condition (1) is met, transistor *Q*_1_ operates in a linear time varying state, and the current flowing through transistor *Q*_1′_s emitter is

(4) $$i_{E}=f\left(V_{B}+v_{RF}+v_{LO}\right)$$

Expression (1) is expanded by the Taylor series, which further yields

(5) $$i_{E}\approx f\left(V_{B}+v_{RF}\right)+f^{\prime }(V_{B}+v_{RF})v_{LO}$$

(6) $$i_{E}\left(t\right)\approx I_{E}\left(w_{RF}t\right)+g_{E}(w_{RF}t)v_{LO}(t)$$

where $I_{E}\left(w_{RF}t\right)$ and $g_{E}(w_{RF}t)$ are periodic functions of the frequency $w_{RF}$ of the RF signal; then, the emitter current *i_E_* of transistor *Q*_1_ contains the component with frequency $mw_{RF}\pm nw_{LO}$. Among the frequency components, the amplitude of the signal with frequency $w_{RF}-w_{LO}$ is the dominant component. Based on the above theory, the detection mixer completes the spectrum shift of the LO leakage signal and RF signal in the output signal, that is, the down-mixing function.

**Figure 3:**
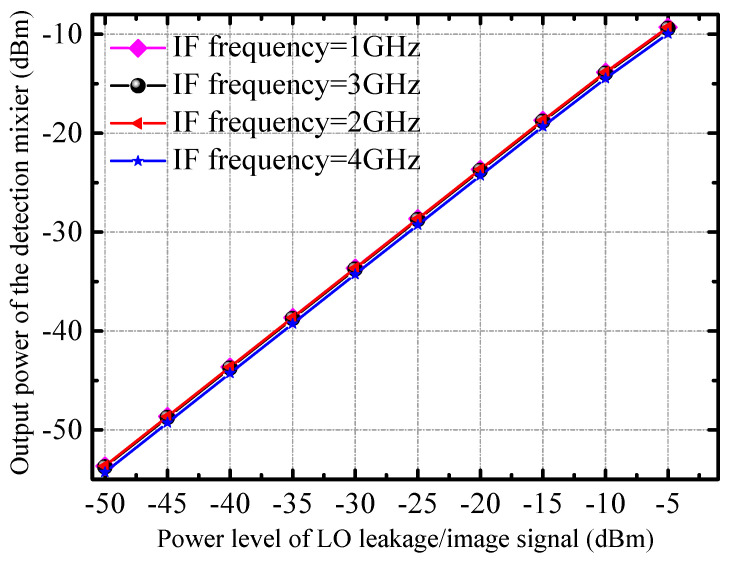
The simulated output power of the DM and the power of the input LO leakage/image signal under different IF frequencies.

Figure 3 shows the simulation results of the detection mixer, the horizontal axis is the power of the LO leakage (or image) signal, and the vertical axis is the power of the IF signal obtained by mixing the RF signal and the LO leakage (or image) signal. When the IF frequency is lower than 5 GHz, the conversion gain (refers to the power ratio of the power of the IF signal output from the detection mixer to the power of the input LO leakage/image signal) of the detection mixer is about −5 dB. It can be seen from the simulation results that the power of the IF signal output by the detection mixer is linear with the power of the LO leakage (or image) signal. Therefore, we can judge the power of the LO leakage signal by the IF output power of the detection mixer. Furthermore, the calibration of the LO leakage signal is completed in combination with the tunable tail current source integrated in the quadrature mixer. The tail current source of the mixer is dynamically adjusted based on the amplitude information of the LO leakage signal, so that the amplitude of the LO leakage signal reaches the minimum value. At this point, the state of the tail current source of the mixer is the state when the LO leakage signal calibration is completed.

### 3.2. Differential Quadrature Mixer

**Figure 4:**
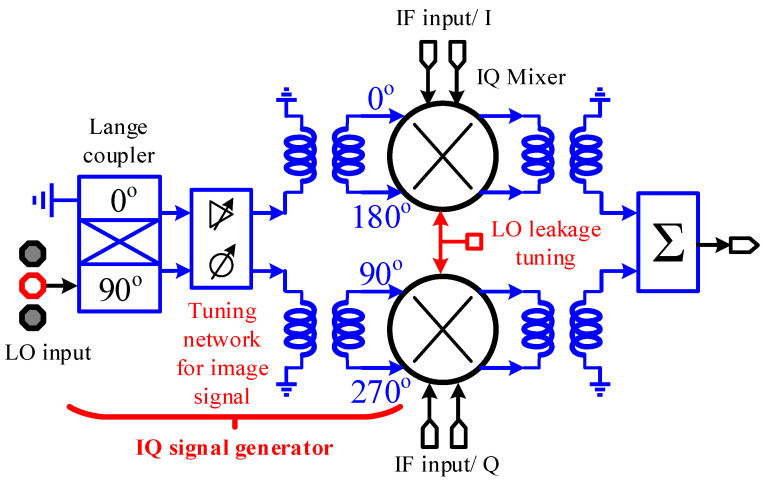
Block diagram of the differential quadrature mixer.

The image rejection IQ mixer is the core component in the zero-IF TX front-end. Figure 4 shows the system block diagram of the image-reject mixer, including a differential IQ signal generation network, two differential mixers with tail current tuning for LO leakage calibration, and a combiner for upper sideband spectrum selection [18].

**Figure 5:**
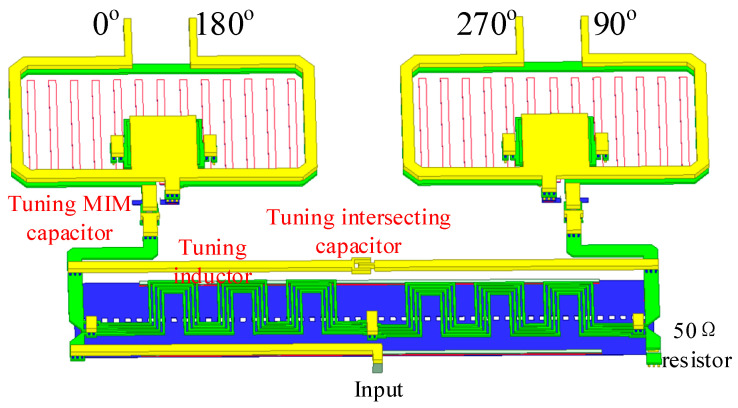
Three-dimensional (3D) view of the IQ signal generator.

**Figure 6:**
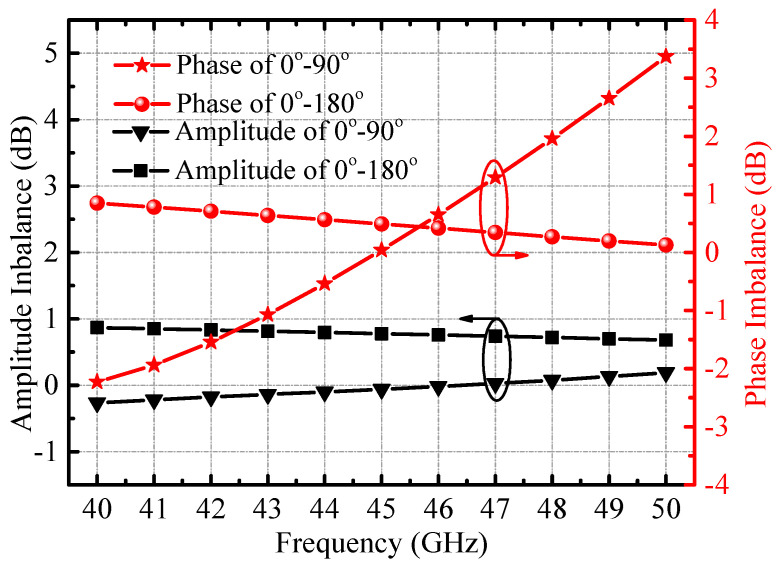
Simulated amplitude and phase balance performance of the IQ signal generator.

The architecture of the image rejection mixer requires four quadrature differential signals (0°, 90°, 180°, 270°), and in order to achieve better image rejection performance, it is necessary to select an appropriate passive circuit to generate differential quadrature signals with better amplitude and phase balance. In order to obtain better amplitude and phase balance in the 40–50 GHz band, an IQ signal generator based on the Lange coupler and transformer balun is used as shown in Figure 5. Unlike our previous reported structures [1], an amplitude and phase balance tuning network between the coupler and balun is adopted, consisting of a series of MIM capacitors, parallel tuned inductors, and an intersecting capacitor. The introduction of the amplitude-phase tuning network greatly improves the amplitude and phase balance characteristics of the IQ generator, and it further effectively improves the image frequency suppression characteristics of the quadrature mixer. The simulated amplitude and phase balance performance of the IQ signal generator are shown in Figure 6; in the 40–50 GHz band, we can see that the amplitude difference between the output ports is less than 1 dB, and the phase difference is within 3 degrees. In addition, within the operating frequency band of the IQ signal generator, the S11 at the input port is better than −15 dB. Since the Lange coupler, transformer balun, and tuning network are all passive devices, after electromagnetic simulation verification, the amplitude and phase balance of the I and Q paths are relatively stable.

**Figure 7:**
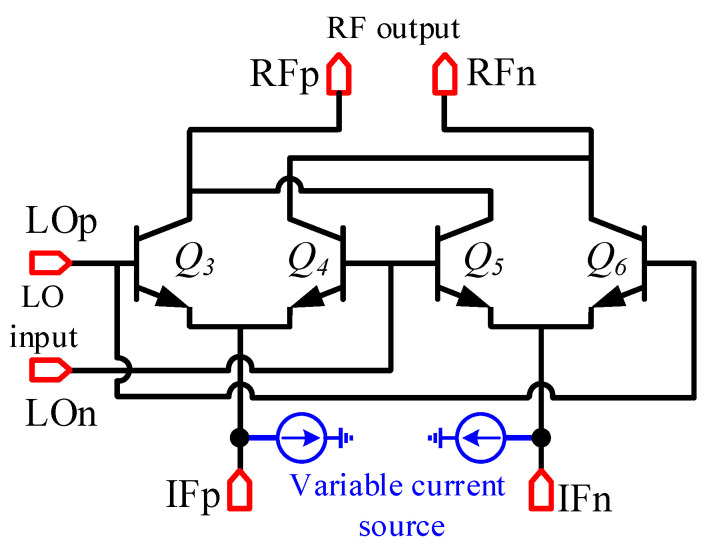
Schematic of the I/Q mixing core in the quadrature mixer.

**Figure 8:**
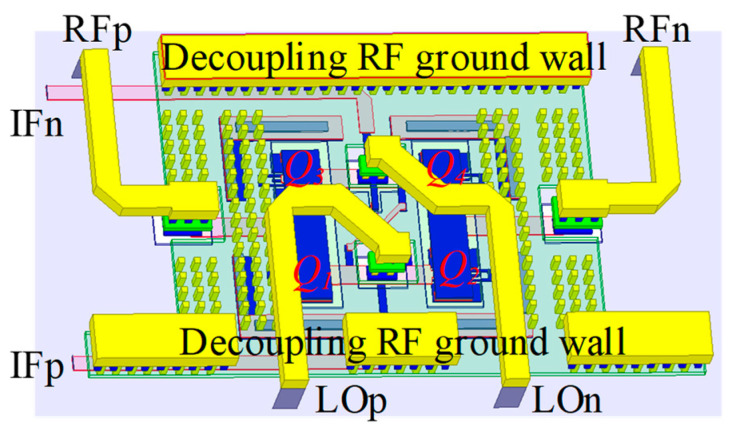
Three-dimensional (3D) layout of the Gilbert mixer core.

The Gilbert cell as depicted in Figure 7 is adopted as the mixing core, and an image-suppressed up-converter consisting of two Gilbert mixers is shown in Figure 3. Gilbert mixers based on a double-balanced structure can achieve good isolation of LO and RF signals, and they have higher conversion gain than single-balanced and passive topologies. The transistors in the mixing core use the dual base and dual collector structure provided in the process to improve the symmetry of the transistor. Unlike traditional active Gilbert mixing cores, this mixing core does not use an active transconductance stage and consists of resistors [19]. The advantage of such a design is that a tunable tail current source can be easily integrated to complete the calibration of the LO leakage of the mixer. The 3D view of the mixing core is shown in Figure 8 with local oscillators and RF signals isolated by a ground wall of metal and via arrays. This effectively improves the isolation between the LO and the RF signal of the mixer [20].

**Figure 9:**
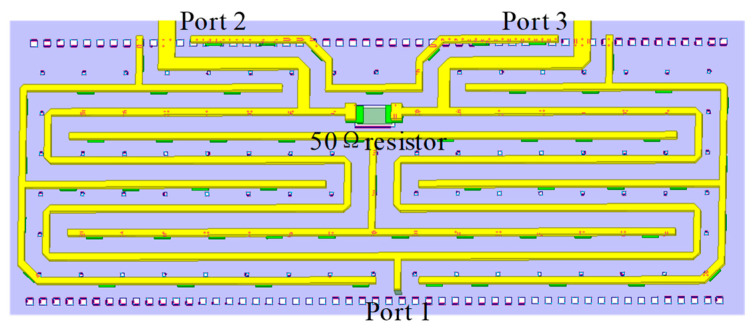
Three-dimensional (3D) layout of the power combiner.

**Figure 10:**
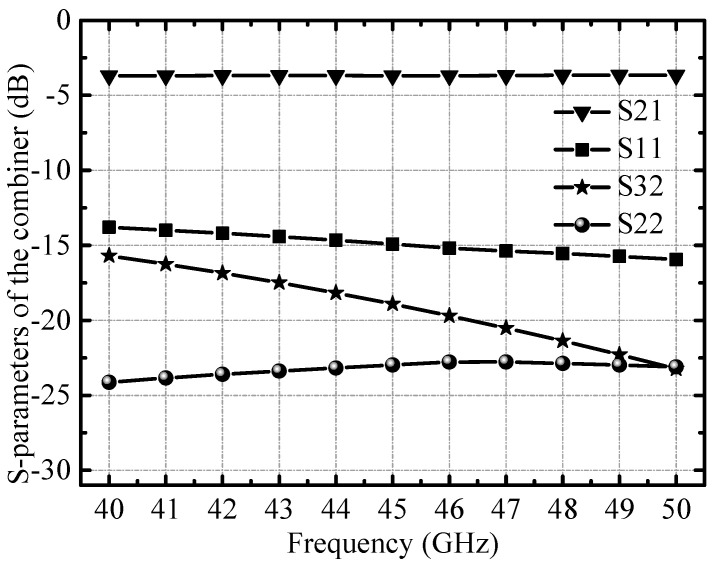
Simulated S-parameters of the power combiner.

The final stage of the IQ mixer uses an on-chip power synthesizer to extract the upper sideband signal. The 3D view of the power combiner is illustrated in Figure 9, and the transmission line is composed of grounded coplanar waveguide (GCPW) transmission lines. The GCPW transmission line is surrounded by RF ground walls on both sides, which can effectively reduce the coupling between adjacent circuits. The simulation results show that in the 40–50 GHz band, the insertion loss of the power combiner is less than 1 dB, and the reflection coefficient of each port is better than −14 dB, as illustrated in Figure 10.

### 3.3. Power Amplifier

**Figure 11:**
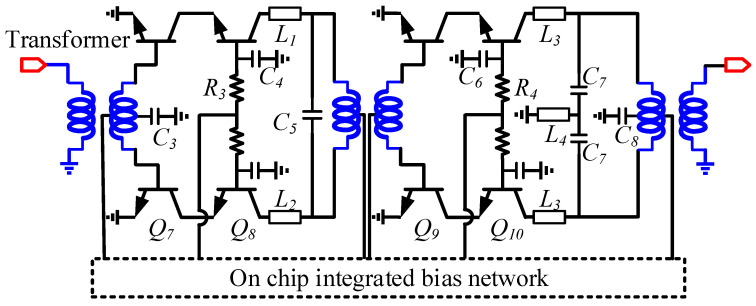
Schematic of the power amplifier.

As shown in the block diagram of Figure 1, the image rejection mixer is followed by a power amplifier, which is mainly used to increase the output power and transmission distance of the TX. The schematic of the power amplifier is shown in Figure 11, consisting of a two-stage differential cascode structure, and the input, output, and intermediate stage matching networks are based on transformers. Due to the limited output power of the image rejection mixers, in order to deliver more than 15 dBm output power, the power amplifier needs to provide a small signal gain close to 20 dB.

**Figure 12:**
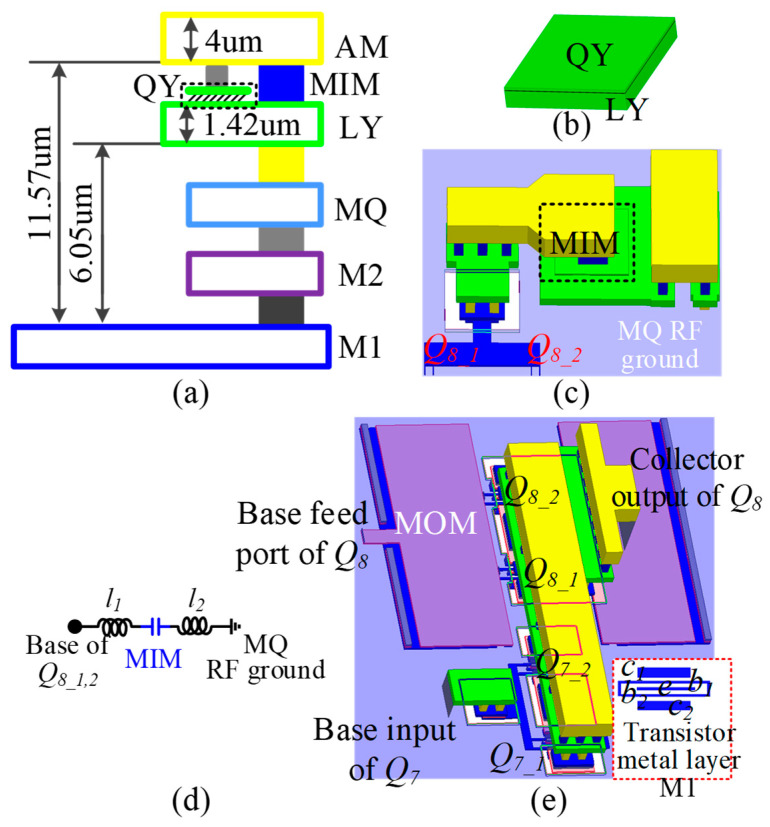
(**a**) Cross-sectional view of the metal layer of the process, (**b**) 3D layout of the MIM capacitor, (**c**) connection of the grounded MIM capacitor for gain boosting, (**d**) equivalent of the connection of MIM capacitor, (**e**) connection of the grounded MOM capacitor for gain boosting.

**Figure 13:**
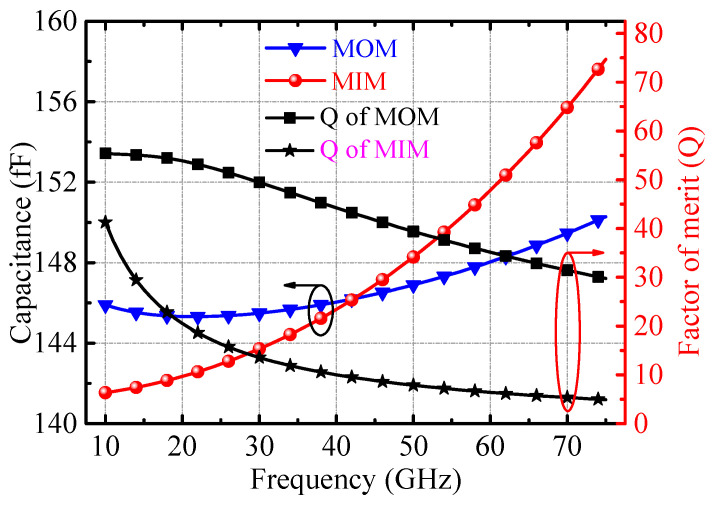
Performance comparison of MIM and MOM capacitor with capacitance around 150 fF.

In the design of the cascode core, we introduce two gain-boosting grounded capacitors (*C*_4_, *C*_6_) as shown in Figure 11 in the common-base transistors [5]. RF ground capacitors in silicon-based processes are mainly composed of metal–insulator–metal (MIM) capacitors or metal–oxide–metal (MOM) capacitors. The metal layer and MIM capacitor of the process used is shown in Figure 12a,b. Limited by the design rules of the process, when using MIM capacitors for gain-boosting, the base of the common-base transistor must first be connected to the top metal level AM and then connected to RF ground through the MIM capacitor. However, the connection inductor shown in Figure 12d had to be introduced across the MIM capacitor, which greatly reduced the Q value of the MIM capacitor. Therefore, the grounded MOM capacitor shown in Figure 12e is used in the design. The MOM capacitor consists of metal layers M1, M2, and MQ in the form of stacked interleaved capacitors, and the capacitor is next to the base of the common-base transistor. As shown in Figure 13, compared with MOM capacitors and MIM capacitors in the 40–50 GHz band, the capacitance value fluctuation range and figure of merit characteristics are significantly improved.

## 4. Experimental Results

**Figure 14:**
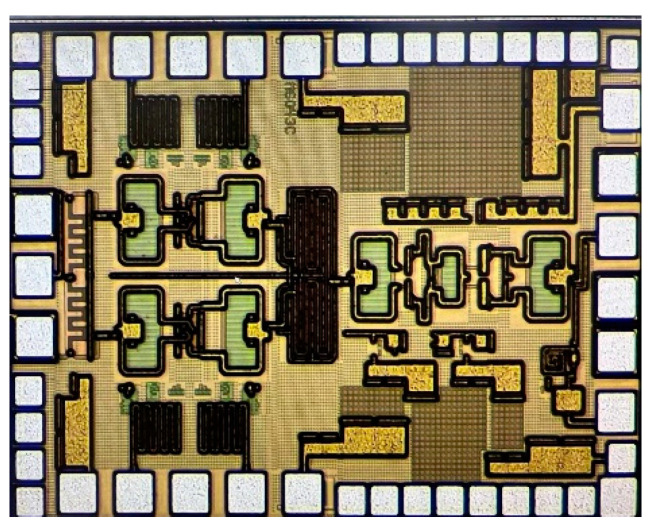
Die micrograph of the 40–50 GHz RF front-end.

**Figure 15:**
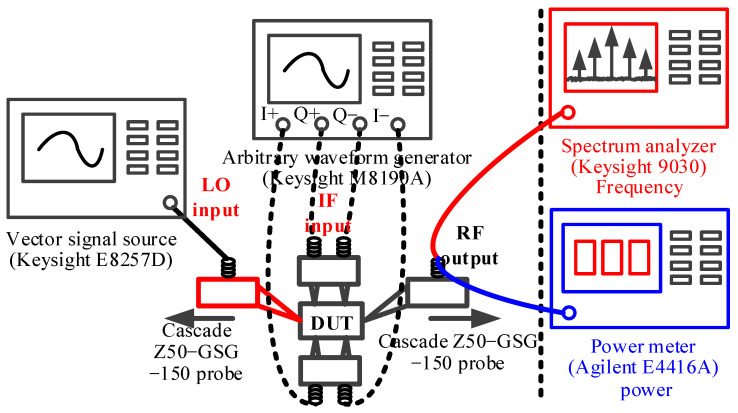
Measurement setup of the 40–50 GHz RF front-end.

The RF front-end is implemented using a 130 nm SiGe BiCMOS process; Figure 14 shows the chip micrograph. The RF front-end including RF and DC PADs consumes a chip size of only 1.15 × 1.5 mm^2^, and the transmitter is biased at 3.3 V power supply. The performance of the chip is performed using a probe station, including an output power 1 dB compression point, amplitude of LO leakage and image signals, and 5 G new radio (NR) signal measurement. The measurement setup of the chip is shown in Figure 15, which mainly includes an output power test and output spectrum test. The IQ IF signals are loaded by an arbitrary waveform generator through the coaxial line to the IF input ports of the chip, and the LO signal is loaded to the LO port through a vector signal source. When performing an output power measurement, a power meter is connected to the RF output for testing. The RF output spectrum is measured by a spectrum analyzer.

**Figure 16:**
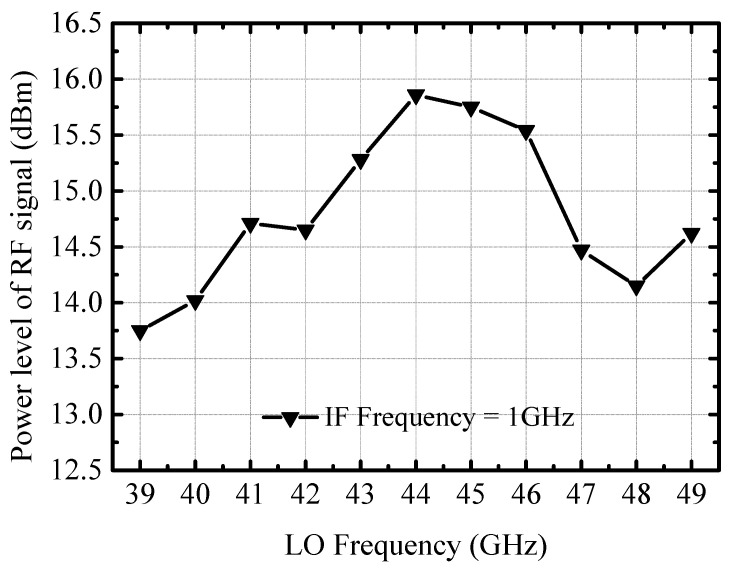
Measured output power 1 dB compression point of the RF front-end.

Figure 16 shows the measured output power 1 dB compression point of the RF front-end with the variation of LO frequency, where the IF frequency is fixed at 1 GHz. The RF output power 1 dB compression point fluctuation in the 40–50 GHz frequency band is less than 2 dB, and the output power is higher than 13.5 dBm. The measured maximum output power 1 dB compression point of the transmitter is about 15.8 dBm at 45 GHz, because the transmitter is designed for the IEEE 802.11aj communication standard, which is defined for communication applications in the 42.3–48.4 GHz frequency band with a center frequency of around 45 GHz.

**Figure 17:**
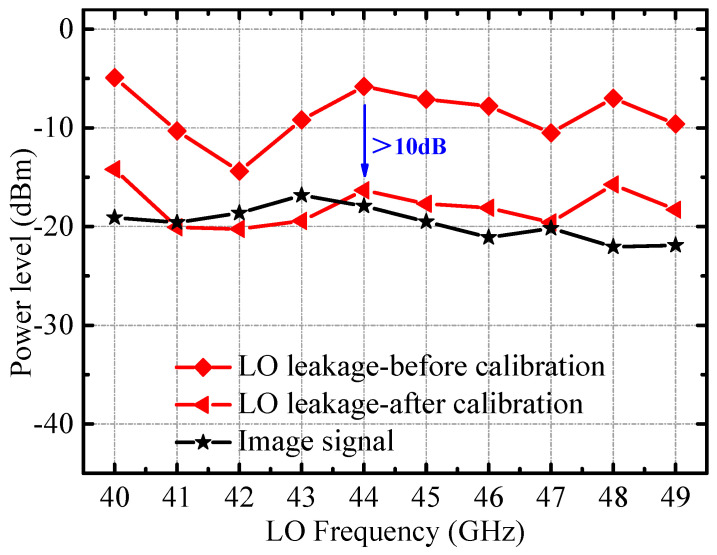
Measured power level of image and LO leakage signals.

**Figure 18:**
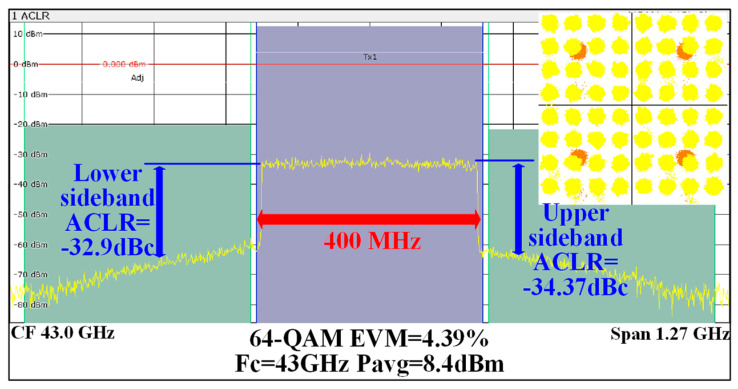
Measured constellation and spectrum of the TX for 64-QAM, 400 MHz bandwidth signal at 43 GHz.

This RF front-end is intended for zero-IF applications, where a poor suppression of LO leakage signals and image signals will greatly deteriorate the modulation performance of the TX. For the image frequency signal, the amplitude and phase tuning network introduced in the IQ signal generator is used to improve the image frequency signal suppression performance of the TX. For the LO leakage signal, the LO signal information detected by the detection mixer is combined with the tunable tail current source integrated in the quadrature mixer to improve the suppression performance of the LO leakage signal. Figure 17 shows the amplitude values of the image frequency signal and the LO leakage signal measured at the RF output after calibration. After calibration, the amplitude values of the image frequency and LO leakage signal are about −20 dBm. Combining Figure 16 and Figure 17, both the image frequency signal and LO leakage signal suppression of the transmitter exceed 30 dBc. In particular, the amplitude of the LO leakage signal drops by nearly 10 dB, owing to the integration of the detection mixer at the RF output. Combining Figure 16, Figure 17 and Figure 18, it can be deduced that in the 40–50 GHz frequency band, the suppression of the LO leakage signal and the suppression of the image frequency signal of the RF output port all exceed 30 dBc.

## 5. Conclusions

Based on a 130 nm SiGe BiCMOS process, this article introduces a zero-IF RF front-end with operating frequencies covering 40–50 GHz. By introducing amplitude and phase tuning networks into the IQ signal generator in the quadrature mixer, the image signal can be calibrated so that the image signal rejection exceeds 30 dBc over the full frequency band. Based on the detection mixer integrated at the RF front-end and the tunable tail current source in the quadrature mixer, this TX enables calibration of the LO leakage signal. After calibration, the TX can suppress the LO leakage signal in the full frequency band by more than 30 dBc.
